# A clinical trial comparing Lanconone® with ibuprofen for rapid relief in acute joint pain

**DOI:** 10.1186/s13063-016-1268-6

**Published:** 2016-04-06

**Authors:** Robert N. Girandola, Shalini Srivastava, Costas C. Loullis

**Affiliations:** Department of Biological Sciences, University of Southern California, Greater Los Angeles, CA USA; Research & Development, Enovate Biolife, Wilmington, DE USA; ANIXIS Biomedical Consulting, Chapel Hill, NC USA

**Keywords:** Ibuprofen, Joint pain, Rapid pain relief, Visual analogue scale, Analgesic, Health supplement, Glucosamine, Nonsteroidal anti-inflammatory drug, Arthritis, Trauma

## Abstract

**Background:**

To study the effect of Lanconone® (1000 mg) on acute pain on exertion as compared to the standard of care, Ibuprofen (400 mg).

**Method:**

The study recruited 72 subjects diagnosed with mild to moderate knee joint pain on exertion. Subjects with Pain Visual Analogue Scale of more than 40 mm were included. Uphill walking was provided as the stressor using Naughton’s protocol on a treadmill. The subjects walked for 10 minutes continuously followed by a rest period and baseline pain score for index knee joint was recorded. Subjects were administered a single dose of Lanconone® (1000 mg)/Ibuprofen (400 mg). Thereafter the same stressor was provided at 0.5, 1, 2, 3, 4, and 6 hours, subsequently, pain scores were recorded on a visual analogue scale. Double stopwatch method was used to evaluate the onset of pain relief and time taken to meaningful pain relief.

**Result:**

Both Lanconone® and Ibuprofen showed the first perceived pain relief at **65.31 ± 35.57** mins as compared to **60.82 ± 32.56** mins respectively. The mean time taken to experience meaningful pain relief in Lanconone® group was **196.59 ± 70.85** mins compared to **167.13 ± 71.41** mins amongst Ibuprofen group. The meaningful pain relief continued for 6 hours.

**Conclusion:**

The current study successfully demonstrated rapid pain-relieving potential of Lanconone® which was comparable to Ibuprofen. No adverse event related to the interventions was reported in the study.

**Trial registration:**

Clinical trials.gov NCT02417506. 21 January 2015.

**Electronic supplementary material:**

The online version of this article (doi:10.1186/s13063-016-1268-6) contains supplementary material, which is available to authorized users.

## Background

A Center for Disease Control (CDC) survey in 2010–2012 showed the prevalence of joint pain to be 22.7 % for adults in the USA. The prevalence of musculoskeletal disorders is 23.9 % for women and 18.6 % for men. Furthermore, recent World Health Organization statistics for the year 2010 reported a 45 % increase in disability due to musculoskeletal disorders during the past decade [1]. The CDC has also projected that 25 % of the world population would be affected by musculoskeletal disorders by the year 2030 [2]. These statistics present only a partial picture because they do not take into account the contribution of risk factors associated with obesity, sedentary lifestyle, sports-related injury, and nutritional deficiencies leading to these disorders.

Pain and debilitation are the central hallmarks of musculoskeletal disorders [3]. Complete recovery from joint disease poses a significant challenge to patients and healthcare providers. Pain plays a major role, as illustrated by a study conducted amongst basketball players where almost 19 % of athletes never resumed their game owing to pain caused by injury, thus experiencing suboptimal postoperative recovery [4]. Similar results were observed in running, football [5], and other sports [6].

Current regimens for joint pain management include pharmacological, surgical, and alternative therapies. Surgical intervention is generally considered a last resort, in the face of the failure of nonsurgical therapies. Pharmacological therapies include nonsteroidal anti-inflammatory drugs (NSAIDs), opioids, corticosteroids, and other analgesics, all of which carry varying degrees of safety risks.

NSAIDs even though are most popular, their long-term use is frequently reported to be associated with several safety concerns, such as gastrointestinal erosions with or without bleeding, cardiovascular risks and hypertension [7–9]. Additionally, increased risk of hypertension as well as blood glucose destabilization was observed in older patients [10]. These safety issues fuel patients’ desires as well as the scientific community’s quest for safer and effective alternative treatments.

Lanconone® (Enovate Biolife, Wilmington DE, USA) is a proprietary botanical active compound, which has demonstrated significant reduction in joint pain in moderate to severe symptoms of osteoarthritis (OA). In an earlier pilot study [11], use of Lanconone® for chronic pain over the period of 12 weeks was investigated. However, reports of rapid pain relief from subjects taking Lanconone® prompted us to confirm these initial findings with a new study. Presently, there are no marketed herbal products with demonstrated analgesic activity in the temporal range of NSAIDs. The present double-blind, comparator-controlled randomized clinical study was designed specifically to evaluate the efficacy of Lanconone® in acute pain, intensity, and onset of pain relief, against ibuprofen as a NSAID comparator.

## Methods

### Subjects

The trial was conducted at the outpatient clinics of two orthopedic surgeons having a regular inflow of subjects with knee pain. Knee joint pain is an appropriate model to study pain relief, as the structural changes here are representative of a stable condition to study acute pain. A total of 72 males and females between 40 and 60 years of age with mild to moderate degenerative changes of the knee joint were randomized in the study.

### Subject inclusion criteria

1. History of moderate to severe knee pain on minimal exertion but no pain at rest.
2. A score ≥40 mm on the pain visual analog scale (VAS) after walking briskly at a pace of 4 ± 0.5 mph on a treadmill without elevation for 10 minutes continuously.
3. Grade II and III joint functionality assessed clinically as per American Rheumatology Association (ARA) classification [12] and radiologically as per Kellgren Lawrence (KL) classification [13].
4. Physician and subject global assessment of joint pain as “poor” or “very poor” after walking briskly at a pace of 4 ± 0.5 mph on a treadmill without elevation for 10 minutes.

### Subject exclusion criteria

1. Any other form of arthritis except OA.
2. Neurological origin of pain, limb deformity, or any other systemic illness that might interfere with the outcome of the study.
3. Subjects taking intra-articular or oral steroids/hyaluronic acid/parenteral NSAIDS for a considerable period.
4. Subject with signs of local lower limb(s) injury.

All subjects provided their well-informed written consent for participation in the study which was recorded audio-visually. The study was conducted in compliance with International Conference on Harmonisation - Good Clinical Practice (ICH-GCP) guidelines. Approval for the study was granted by Independent Ethics Committee (IEC-Aditya registered with the Office for Human Research Protections in the US Department of Health and Human Services under registration number IRB00006475). The trial was registered at clinical trials.gov under registration number NCT02417506.

### Study design

**Fig. 1:**
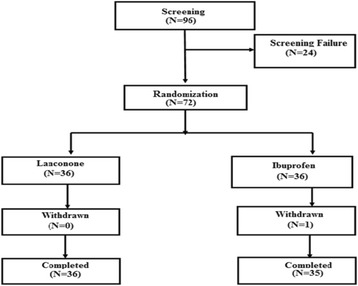
Participant flow in the study

This study was a prospective noninferiority, randomized, double-blind, comparator-controlled, parallel group, multicenter clinical trial designed to assess the effects of Lanconone® in acute pain. The subjects were randomized to one of the two treatment groups, with appropriate blinding maintained for the subjects, the study coordinators, as well as the investigators. Figure 1 shows the flow of participants in the study.

### Recruitment

During the screening, the subjects were assessed for the history of pain upon exertion but no pain at rest. They were then asked to walk briskly at a pace of 4 ± 0.5 mph on a treadmill without elevation for 10 minutes, following which they marked the level of pain on the VAS (0 being no pain and 100 being unbearable pain).

Subsequently, a clinical examination was done by the investigator to determine functionality based on ARA classification, defined as pain in the knee joint on most days, crepitus on motion, morning stiffness ≥30 minutes, age >50 years, and bony enlargement on palpation [14]. Subjects fulfilling four out of five criteria were included in the study.

A radiographic image of the index knee joint (refers to the joint of each subject having the higher VAS score) was also obtained. The X-ray image was graded for KL scale [15] by a radiologist and confirmed by the orthopedic surgeons. Subjects graded by the investigators in grade II (defined as small osteophytes, possible narrowing of the joint) and grade III (defined as multiple, moderately sized osteophytes, definite joint space narrowing, some sclerotic areas, possible deformation of bone ends) were enrolled in the study.

A global assessment of knee joint pain by the investigator and subject was taken, expressed as “very good”, “good”, “fair”, “poor”, and “very poor”. The subjects with “poor” and “very poor” assessments were included in the study.

Subjects satisfying all of the inclusion criteria were entered into the study. Once the subjects qualified at the screening visit, a washout period of 7 days was ensured prior to the study start for those on analgesics for knee pain.

### Study procedure

On the day of randomization, subjects were again asked to walk briskly at a pace of 4 ± 0.5 mph on a treadmill without elevation for 10 minutes to assess the worsening of pain during the washout period. Knee pain was assessed by the investigator and the subject. An uphill walking protocol is considered most appropriate to induce knee pain which would be close to real-life situations and can be applied to pain in day-to-day wear and tear [16]. A modified Naughton’s protocol [17] of uphill walking on a treadmill was provided as the stressor. Subjects were initially familiarized with the treadmill usage protocol. All instructions were provided clearly to the subjects. After an initial resting time of 10 minutes, subjects were asked to stride the treadmill belt. The treadmill speed was set up to 1 mph with 0° incline for the first 2 minutes followed by an increase of speed to 2 mph which remained constant thereafter. The inclination increased by 3.5 % every 2 minutes. Subjects walked on the treadmill for 10 minutes and marked their baseline pain intensity using the VAS within 5–10 minutes of ending the walking protocol. The time gap was provided to avoid muscle fatigue-related high scores. They then rested until the next VAS time point.

Immediately after the baseline pain was recorded, subjects were administered a single oral dose of two capsules of Lanconone® or ibuprofen.

Pain VAS scores were assessed at 0.5, 1, 2, 3, 4, 5, and 6 hours post dosing. The subject walked for 10 minutes prior to all assessment time points, followed by rest for 50 minutes. The subjects rated their pain on VAS scale 5–10 minutes following completion of the treadmill exercise as per specified protocol.

#### Treatment

Both Lanconone® and ibuprofen were prepared in orange-colored capsule size 0 and matched in weight and color. Each 500 mg capsule of Lanconone® (Enovate Biolife) contained *Oroxylum indicum*, *Withania somnifera*, *Zingiber officinale*, *Commiphora mukul*, *Smilax china*, *Pluchea lanceolata* and *Boswellia serrata*. The matched capsule of ibuprofen (HuBei Granules-biocause Pharmaceutical Co. Ltd, Yangwan Road, Jingmen, Hubei, China) contained 200 mg of the active ingredient plus excipients. Both Lanconone® and ibuprofen were prepared under Good Manufacturing Practices. Ibuprofen was selected as the comparator for the study because of its high efficacy to reduce musculoskeletal pain, its common use as an anti-inflammatory drug, and its better safety profile amongst NSAIDs. The study subjects were administered two capsules of either Lanconone® or ibuprofen. Being a single dose study, 100 % compliance with both interventions was assured.

#### Pain VAS

The pain VAS has been effectively used to measure acute as well as chronic pain in degenerative joint conditions [18] and has demonstrated sensitivity to changes in pain assessed hourly following analgesic therapy [19]. On a scale of 100 mm subdivisions, 0 indicated no pain and 100 the highest pain. Subjects were asked to rate the severity of their pain at the screening visit as well as at baseline and at various time points such as 0.5, 1, 2, 3, 4, 5, and 6 hours. The pain intensity difference was calculated by comparing pain VAS scores at baseline with each post-treatment time point.

#### Onset and time to meaningful pain relief

A “double stop-watch method” was used to determine first perceived pain relief (reduction in VAS pain by 10 mm, as perceived by the subjects compared with the recently scored baseline) and time to meaningful pain relief (reduction in VAS pain by 40 mm, as perceived by the subjects compared with the recently scored baseline), thus defining the precise onset of the drug action. Immediately after dosing, the subjects were given two stop watches and instructed that once they started perceiving the pain relief for the first time, they were to switch off one of the two stop watches and hand it to the study coordinator. Similarly, when they experienced meaningful pain relief they were to switch off the second stop-watch. The study coordinator recorded both time points for pain relief [20].

### Safety assessment

The subjects reported to the investigator any adverse events during the study duration, and the adverse event was recorded by the investigators. The investigator also sought causality for each reported adverse event.

### Quality assurance

The study was conducted in compliance with the ICH-GCP guidelines laid down in E6 (R1) as per a preapproved monitoring and auditing plan by a team independent of the investigational site (Vedic Lifesciences, Redwood, CA, USA).

### Statistical analysis

A randomized treatment list was generated for assigning each subject to one of two treatment groups. All analyses were performed using SPSS® software version 10.0 (IBM Corporation, 1 New Orchard Road, Armonk, New York 10504-1722, United States), using a chi-square test for categorical data and analysis of variance and Student’s t tests (independent unpaired two-sample test) for continuous variables. The accepted level of significance was α ≤0.05.

## Results

### Demographics

Age, height, and weight are presented in Table 1. There were no significant differences between groups for age (*p* = 0.14) and weight (*p* = 0.74). There was a significant difference (*p* <0.05) between groups for height (*p* = 0.05). The mean difference in height was in the order of 4 cm; however, this did not have significant impact on body mass index (*p* = 0.08) and therefore the study outcome was not affected by this difference. The subjects recruited were 18 males and 53 females. However, gender distribution in both groups was matched (*p* = 0.24).

**Table 1:** Demographics

Parameter	Lanconone®	Ibuprofen	*p* value^a^
Number of cases	36	35	
Age (years)			
Mean	49.56	51.60	0.14
Standard deviation	06.26	05.28	
Range	40.00–62.00	41.00–59.00	
Height (cm)			
Mean	153.19	157.27	0.05*
Standard deviation	07.27	09.67	
Range	142.00–169.00	144.00–182.00	
Weight (kg)			
Mean	65.61	64.71	0.741
Standard deviation	11.20	11.66	
Range	48.00–93.00	45.00–92.00	
Body mass index (kg/m^2^)			
Mean	27.8	26.19	0.079
Standard deviation	4.15	3.93	
Range	19.68–39.21	18.26–37.66	

^a^Student’s *t* test

*p <0.05 - Statistically significant

*p* >0.05 - Not significant

### Joint health

All subjects included in the study had no pain at rest. Both groups were matched in their radiological and functionality assessment at baseline.

### Pain VAS score difference

**Fig. 2:**
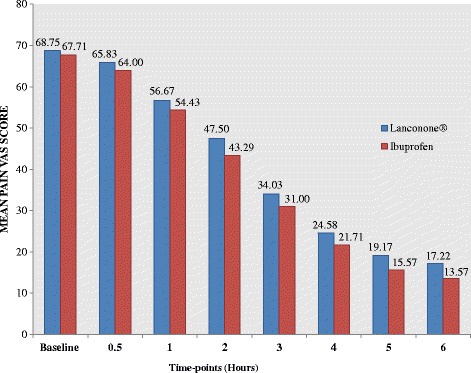
Pain reduction with single dose of Lanconone® versus ibuprofen over 6 hours. *VAS* visual analog scale

At baseline, the mean pain VAS score was 68.75 ± 10.58 for the Lanconone® group and 67.71 ± 12.85 for the ibuprofen group (*p* = 0.71). Pain VAS scores for both groups declined over 6 hours with significant pain relief seen in both groups (*p* <0.05), starting at 0.5 hours, as shown in Fig. 2. There were no significant differences between groups at any time points, demonstrating equivalence between Lanconone® and ibuprofen.

#### Mean pain intensity difference

At 0.5 hours, the mean pain VAS intensity showed a significant fall of 4.2 % for the Lanconone^®^ group and fall of 5.5 % for the ibuprofen group compared to baseline. The same trend was observed from 1 to 5 hours. At 6 hours, the mean pain VAS score in Lanconone® subjects showed a significant fall of 75.0 % and that for ibuprofen subjects was 80.0 %. The pain intensity difference for the Lanconone® group followed a downward trend similar to ibuprofen as shown in Table 2.

**Table 2:** Pain intensity difference

Duration (hours)	Pain intensity difference		*p* value^a^
	Lanconone®	Ibuprofen	
0.5	–2.92 ± 04.20	–3.71 ± 05.05	0.477
1	–12.08 ± 09.59	–13.28 ± 11.37	0.632
2	–21.25 ± 11.04	–24.42 ± 16.08	0.337
3	–34.72 ± 14.04	–36.71 ± 16.76	0.590
4	–44.17 ± 17.55	–46.00 ± 15.66	0.644
5	–49.58 ± 14.31	–52.14 ± 14.31	0.454
6	–51.53 ± 15.85	–54.14 ± 12.88	0.448

^a^Analysis of variance

#### Study responders

**Fig. 3:**
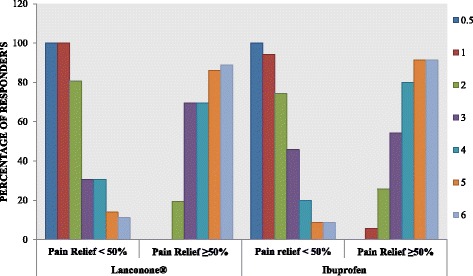
Distribution of responders across the Lanconone® and ibuprofen groups

Subjects demonstrating an overall ≥50 % decrease in pain intensity were termed responders. The majority of study subjects fell into this category between 2 and 6 hours after administration of interventional products. At the end of 2 hours, 19.4 % of subjects in the Lanconone® group showed ≥50 % pain relief as compared with 25.7 % of subjects in the ibuprofen group. The percentage of responders rose to 69.4 % in the Lanconone® group as compared with 80.0 % in the ibuprofen group from 3 to 5 hours. At the end of 6 hours, 88.9 % of the Lanconone® subjects showed ≥50 % pain relief as compared with 91.4 % for the ibuprofen subjects, with no significant difference between the groups (*p* >0.05). These results are shown in Fig. 3.

### First perceived pain relief

The mean time taken to perceive pain relief of at least 10 mm on the VAS from baseline by the Lanconone® group was found to be 65.31 ± 35.57 minutes as compared with 60.82 ± 32.56 minutes in the ibuprofen group. The two groups were not significantly different in this respect (*p* >0.05).

### Meaningful pain relief

The mean time taken to experience meaningful pain relief (a VAS scale value reduction of 40 mm) in the Lanconone® group was 196.59 ± 70.85 minutes compared with 167.13 ± 71.41 minutes amongst the ibuprofen group. The two groups were not significantly different in this respect (*p* >0.05).

### Safety results

As shown in Table 3, three subjects in the Lanconone® group had adverse events as compared with one subject in the ibuprofen group. All of the adverse events were deemed to be not related to either of the products by the investigators.

**Table 3:** Safety results

Adverse event	Lanconone®	Ibuprofen	*p* value^a^
	(*n* = 36)	(*n* = 35)	
Mild giddiness	2	0	
Vertigo	1	0	
Breathlessness	0	1	
Total number of subjects	3	1	0.303

^a^Chi-square test

## Discussion

Pain is a common complaint for all individuals in different stages of life. Musculoskeletal pain, being the leading and the most debilitating condition, is a major cause of concern for physically active people. The present study was undertaken to compare the kinetics of pain relief from Lanconone® 1000 mg versus ibuprofen 400 mg in a painful knee joint. Several studies have proven ibuprofen to be efficacious in the management of pain in subjects with joint pathology [21, 22], albeit with serious side-effect liabilities.

We found no significant difference in the onset and time to meaningful pain relief for the two products studied. Larger studies may be needed to confirm these findings; however, the present study indicates that Lanconone® 1000 mg is as effective as ibuprofen 400 mg in acute musculoskeletal pain. This to our knowledge is the first such demonstration by a botanical dietary supplement.

Ibuprofen has been extensively studied in relation to pain in knee OA [23]. It has been established that pain in the knee joint correlates well with the pathological condition of the knee joint [24]; hence the pain VAS for both groups in our study is comparable because all subjects were in the same stage of OA. Meaningful pain relief is a critical endpoint in pain studies. The American Pain Society has stated that a pain-relieving effect, although statistically significant, is irrelevant if the pain relief is not meaningful to the patients. A score of 40 mm on a 100 mm scale of pain VAS is considered to be clinically significant pain relief [25]. An exploratory study from 2003 stated that clinically meaningful reduction of acute pain was achieved with a 40–45 % decrease on a pain numerical rating scale [26] similar to the pain VAS, where 0 correlated to no pain and 10 to worst possible pain. This outcome allied well with our study, wherein meaningful pain relief was reached at the pain VAS score of 40 mm. The time to reach meaningful pain relief was similar in both groups.

The ingredients of Lanconone® have a vast body of scientific data. *B. serrata* has been studied in various animal and human trials with different inflammatory conditions such as OA and rheumatoid arthritis, and so forth [27]. The in-vitro studies show that it has cyclooxygenase (COX) and 5-lipooxygenase (LOX) inhibitor activity. Also, in a human trial with the use of 30 % acetyl-11-keto-b-boswellic acid (AKBA), a constituent of boswellia resulted in the reduction of matrix metalloproteinase-3 which is a marker for central pain sensitization [28]. *C. mukul* has been studied in an animal model in comparison with ibuprofen and phenyl butazone for its anti-inflammatory effect [29] in OA. *Z. officinale* is hypothesized to block the activity of COX enzymes and leukotrine and prostaglandin synthesis, thus influencing the pain processing mechanism; it has also been studied for its positive effect on exercise-induced muscle pain [30]. *P. lanceolata* has been found not only to be anti-inflammatory but also to have good analgesic properties. A preclinical study has compared the anti-inflammatory effect of *P. lanceolata* with drugs such as ibuprofen and has proven it to be better [31]. Thus, the rapid pain relief by Lanconone® may be due to the combined analgesic effect of these ingredients.

This trial was designed to study the effect of the supplement for pain relief in postexertion acute joint pain. However, Lanconone^®^ may be of benefit in joint pain that exacerbates on exertion irrespective of pathological or non-pathological origin of pain. While this was a study in a nonathletic population, the data warrant further clinical work into the use of Lanconone® as a safer alternative for pain relief in athletes and other populations. Larger, preferably postmarketing, studies may be required to completely rule out potential side effects of Lanconone®. Therefore, based on traditional and modern data [32] on the component ingredients available, it is not unreasonable to propose Lanconone® for long-term use as a potent and rapid action analgesic for acute joint pain.

The pain-relieving effect of Lanconone® could not be studied beyond 6 hours because of methodological and ethical challenges. However, the pain levels in both groups at the end of 6 hours were in the 10–20 range on the VAS scale, indicating that the analgesic effect for 1000 mg of Lanconone® would probably persist for a considerable time beyond 6 hours.

## Conclusion

Lanconone®, a proprietary botanical active compound, was proven to be efficacious for joint health on chronic administration in an earlier randomized placebo-controlled trial. The present study demonstrated rapid pain relieving potential of Lanconone® which is comparable with that of Ibuprofen, one of the most widely used NSAIDs.

Lanconone® can thus be used as a safe standalone short-term and long-term analgesic, as well as a good complement to potent chondroprotective agents and anti-inflammatory supplements, which are presently facing controversies not only regarding their role in pain and inflammation [1] but also their role in cartilage rebuilding [33].

## CONSORT Statement

The current manuscript adheres to “Consolidated Standards of Reporting Trials” guidelines. The compliance to the guidelines has been demonstrated in Additional file 1.

## Additional file

Additional file 1: CONSORT checklist. (DOCX 65.5 kb)

## Abbreviations

AKBA: Acetyl-11-keto-b-boswellic acid
ARA: American Rheumatology Association
CDC: Center for Disease Control
COX: Cyclooxygenase
KL: Kellgren Lawrence
LOX: Lipooxygenase
NSAID: Nonsteroidal anti-inflammatory drug
OA: Osteoarthritis
VAS: Visual analog scale
